# Physical activity and addressing emotional needs can help reduce medication dependence in autism

**DOI:** 10.3389/fpsyg.2025.1509827

**Published:** 2025-11-14

**Authors:** Mahbod Sahebi, Majid Tamdjidi, Jesper Thorell, Parisa Azizi

**Affiliations:** 1 Hermods AB Yrkeshögskola, Umeå/Östersund, Sweden; 2 Frösunda Omsorg AB, Stockholm, Sweden; 3 Hässelby-Vällingby Stadsdelsförvaltning, Stockholm, Sweden; 4 VC Ängshavrebackens Gruppboende, Stockholm, Sweden; 5 Avdelningen För Vuxna, Boende Vuxen LSS Och Socialpsykiatri, Hässelby-Vällingby stadsdelsförvaltning, Stockholm, Sweden

**Keywords:** autism spectrum disorder (ASD), physical activity/exercise interventions, pharmacological treatments, medication dependence, emotional/social outcomes

## Abstract

Autism spectrum disorder (ASD) is a neurodevelopmental disorder that causes deficits in social interactions, communication skills, intellectual limitations, and self-injurious behaviors. Common systemic comorbidities include gastrointestinal issues, obesity, and cardiovascular disease. Recent study has found a link between gut microbiota alterations and neurobehavioral symptoms in children with ASD. Physical activity and exercise therapy have been demonstrated to promote communication and social interaction while also positively influencing microbiota composition. This research intends to highlight how various sports can improve speech and social abilities in autistic children while also benefitting gut microbiota composition. Parents of autistic children confront several problems. Many parents are too busy or lack the essential information to care for their children at home, so they rely on government-provided healthcare facilities. This condition hinders these youngsters from having a regular upbringing, resulting in behaviors more akin to younger children as they get older. They frequently suffer from mental health concerns and may fail to articulate their needs and desires effectively. The inability to articulate oneself can lead to negative conduct, especially against others who remind them of their parents or siblings. Addressing these issues may entail examining early memories and involving people in activities that match their interests and developmental phases. By doing so, they may feel more understood and sensitive to instruction, like a small child.

## 1 Introduction

A complex neurodevelopmental disorder known as autism spectrum disorder (ASD) is marked by difficulties with social interaction, communication, and repetitive activities. Since its first discovery in 1943 by Dr. Leo Kanner, autism has come to be seen as a spectrum that includes a diverse range of skills and limitations. Because of this variability, no two autistic people are the same; instead, they all have different perspectives on the world. Comprehending autism is essential not just for developing compassion and understanding but also for driving research and building inclusive settings that promote the full potential of persons with autism.

According to the Centers for Disease Control and Prevention (CDC), 2014, 1 in 68 children have autism spectrum disorder (ASD), a complex developmental disease that causes serious difficulties with behavior, social skills, and communication (American Psychiatric Association, 2013). Classic autism, Asperger's syndrome, Rett's condition, Childhood Disintegrative condition, and Pervasive Developmental Disorder-Not Otherwise Specified are all included under the Diagnostic and Statistical Manual of Mental Disorders (DSM-5) under the category of ASD. In addition to challenging behaviors like self-harm, aggression, and non-compliance, people with ASD frequently display a variety of stereotyped behaviors or interests, such as compulsions, echolalia, and motor stereotypies like hand flapping and body rocking (Bodfish et al., 2000), along with challenging behaviors like violence, self-harm, and non-compliance (Fox et al., 2002; Singh et al., 2006).

People diagnosed with ASD generally exhibit symptoms in three areas, namely deficits in social interaction, communication skills, and motor performance (Edition, 2013), however the clinical manifestations substantially vary by individual and age group (Vandereycken et al., 2016). Usually, a variety of interventions of differing intensities are used to address these maladaptive behaviors. These interventions include physical therapy, occupational therapy, speech-language therapy, and behavioral interventions such as applied behavior analysis (De Boer et al., 2004; Myers et al., 2007).

Up to 79% to 83% of children with ASD struggle to execute age-appropriate motor skills, despite this fact not being one of the major diagnostic criteria of ASD (Green et al., 2009; Hilton et al., 2012). Throughout development and youth, these motor restrictions are seen. Toddlers with ASD have been found to have both fine and gross motor delays (Landa and Garrett-Mayer, 2006); however, it is unclear if this motor delay can be distinguished from overall developmental delay (Provost et al., 2007). Regardless of whether an intellectual handicap is present or not, children with ASD frequently have motor deficits (Bhat et al., 2011). Using a standardized motor test, a group of 101 school-aged children with ASD showed that 97% of them had an intellectual disability (IQ < 70) and 70% had near-normal or normal intelligence (IQ < 70). These children were found to be unable to perform age-appropriate motor skills (Green et al., 2009). Participation in the activities required to foster the development of age-appropriate social, communicative, behavioral, and cognitive abilities may be restricted due to challenges executing age-appropriate motor skills (Bhat et al., 2011).

According to Srinivasan, motor deficits can also make it more difficult to engage in the physical activity required to support optimum health and wellbeing (Srinivasan et al., 2014). In comparison to their peers who are usually developing, children and adolescents with ASD have lower levels of physical activity (Mccoy et al., 2016) and are more likely to be overweight or obese (Mccoy et al., 2016; De Vinck-Baroody et al., 2015). Due to unique motor impairments or variations in other domains that impact how they learn motor skills, children with ASD may struggle to achieve age-appropriate motor abilities (Moraes et al., 2017). Age-appropriate motor abilities may be directly impacted by the deficiencies in postural control, motor imitation, and motor planning that children with ASD exhibit (Downey and Rapport, 2012).

Furthermore, according to Tomcheck and Dunn up to 90% of childs with ASD have sensory processing issues (Tomchek and Dunn, 2007). These issues can include tactile hypersensitivity and other impairments in sensory modulation, which can make it difficult for them to continue engaging in motor activities long enough to acquire the age-appropriate motor skills (Schauder and Bennetto, 2016; Robertson and Baron-Cohen, 2017).

Certain tactics can assist children diagnosed with ASD gain new motor skills and manage their distinct social communication and behavior patterns. Important strategies including: Motor Skills, Make use of regular routines, visual aids, and organized practice; Use interest-based learning, behavioral interventions, and social storytelling to promote social communication and conduct; Feedback and Guidance, offer encouraging words, precise directions, and role models. These techniques help childs with ASD develop and function better by creating a supportive atmosphere that is suited to their requirements.

Three systematic evaluations have examined the impact of motor or exercise interventions on the motor outcomes of children diagnosed with ASD (Dillon et al., 2017; Healy et al., 2018). A drawback of these evaluations is that, in order to determine the overall impact of motor intervention on motor outcomes, they merged data from several distinct intervention types, including physical education, aquatic therapy, and hippocratesis. Despite this, all reviews noted improvements in motor outcomes. Educators, physicians, and researchers may benefit more from knowing how different kinds of motor therapies affect certain motor outcomes in childs with ASD. The earlier evaluations' failure to identify the methods employed in the research to enhance learning in childs with ASD is another drawback. In order to fill these gaps in the literature, this systematic review provides evidence-based data that can be used to guide the choice of appropriate motor interventions to improve particular motor outcomes in children with ASD and the choice of appropriate strategies to support motor learning in children with ASD.

This systematic review's main goal is to assess the data about how motor and physical activity therapies affect children with ASD's motor outcomes and to compare the long-term effects of physical activity and medication on individuals with autism.

For those with autism spectrum disorder (ASD), physical activity has been demonstrated to have a variety of advantages, such as enhanced motor abilities, social connections, and behavioral results. Crucially, taking part in regular physical activity might help lessen the need for medication to control the symptoms of ASD. Numerous researches have demonstrated the beneficial effects of physical activity on lowering maladaptive behaviors and enhancing general wellbeing in people with ASD (Zhang et al., 2020). For example, studies have linked structured exercise programs, martial arts, and swimming to less anxiety, better moods, and improved social skills. These advancements may lessen the need for pharmaceutical therapies, which are frequently recommended to treat behavioral and emotional issues in people with ASD. Furthermore, it has been shown that exercise improves drug metabolism, which may enable lesser dosages of medication to have the same therapeutic benefits (Sefen et al., 2020). This can be especially helpful in reducing the negative effects of taking medications for an extended period of time. Emerging research highlights gut microbiota dysbiosis as a potential modulator of ASD symptoms, with exercise shown to positively influence microbial diversity (Sefen et al., 2020). This suggests a bidirectional pathway where physical activity may alleviate both behavioral and systemic comorbidities. This narrative review synthesizes evidence from peer-reviewed studies (2000–2024) identified through systematic searches of PubMed, Scopus, and PsycINFO.

### 1.1 Inclusion criteria

Human subjects with ASD diagnosis; Measurable outcomes (behavioral, physiological, or social); Both intervention and observational studies.

Data extraction and synthesis:

Studies were analyzed thematically to compare:

1. Efficacy of physical activity vs. pharmacological interventions
2. Reported side effects and long-term outcomes
3. The role of family/emotional support in treatment adherence

This review has three key objectives:

1. To systematically compare physical activity and pharmacological interventions for ASD symptom management
2. To evaluate the evidence for exercise as a medication-sparing strategy
3. To provide clinical recommendations for integrating emotional support with physical interventions

The findings aim to guide clinicians, caregivers, and policymakers toward more holistic ASD management approaches.

### 1.2 Evidence from literature

Recent meta-analyses confirm that structured exercise programs reduce ASD-related stereotypy by 20-30% (Sefen et al., 2020), while pharmacological interventions show comparable short-term efficacy but higher adverse effects (Aishworiya et al., 2024).

#### 1.2.1 Clinical perspective

This review prioritized meta-analyses and randomized controlled trials where available, but also included seminal observational studies to capture broader perspectives.

This review also argues for prioritizing physical activity as a first-line intervention, reserving medication for acute symptom management.

## 2 Methodology

### 2.1 Search strategy

Systematic searches in PubMed/Scopus (2000–2024) combined terms: (autism^*^ OR ASD) AND (exercis^*^ OR “physical activit^*^”*)*.

### 2.2 Selection criteria

*Included studies measured behavioral/physiological outcomes in ASD populations with control groups*.

### 2.3 Data synthesis

**Figure 1:**
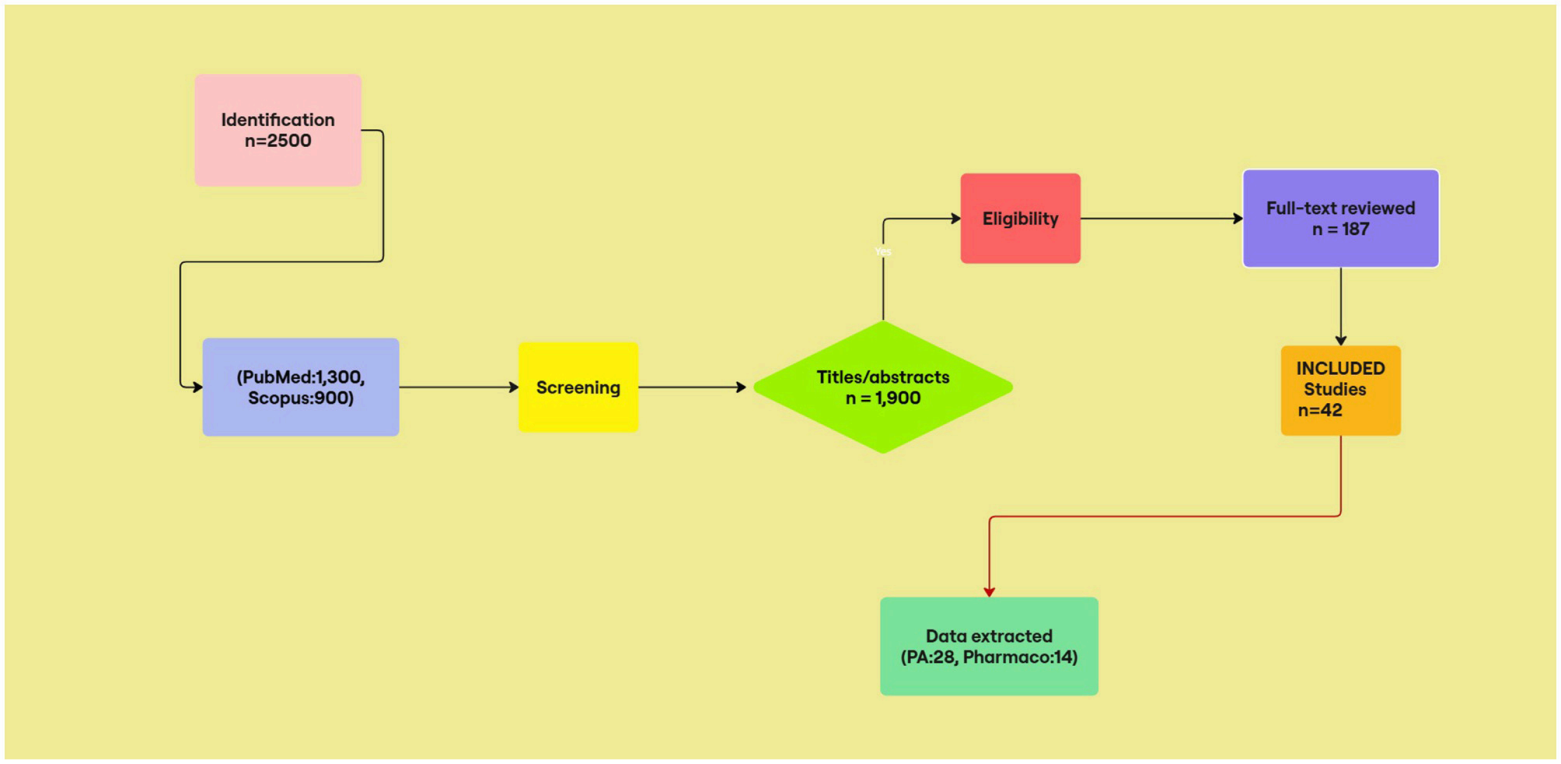
PRISMA 2020 flow diagram of study selection. Initial searches yielded 2,500 records. After screening titles/abstracts (*n* = 1,900) and assessing 187 full-text articles, 42 studies were including (28 physical activity, 14 pharmacological).

Findings were thematically analyzed; 42 of 2,500 screened records met criteria (Figure 1, PRISMA flow diagram).

#### 2.3.1 Thematic analysis methodology

Studies were categorized by intervention type (physical activity vs. pharmacological), with outcomes analyzed for efficacy, side effects, and adherence.

## 3 Results

### 3.1 Standard treatment of ASD

The goal of ASD therapy is usually to inhibit or eradicate maladaptive behavioral tendencies including rigidity and stereotyped movements while promoting cognitive, linguistic, and social development (Koenig et al., 2010).

The conventional approach to treating autism spectrum disorder (ASD) usually includes a mix of individualized interventions. Here are a few typical methods:

#### 3.1.1 Behavioral approaches

They concentrate on altering behavior by comprehending the pre- and post-behavior processes. A well-known technique that promotes desired behaviors and inhibits undesirable ones is called Applied Behavior Analysis (ABA; Gulsrud and Renno, 2021).

#### 3.1.2 Developmental approaches

They are designed to enhance particular developmental capacities, such language or motor skills. Occupational therapy and speech and language therapy are typical examples (Gulsrud and Renno, 2021).

#### 3.1.3 Therapeutic education

For childs with ASD, structured educational programs can be quite beneficial. Individualized education plans (IEPs), which address each child's specific learning requirements, are frequently a part of these programs (Gallin et al., 2024).

#### 3.1.4 Social-relational approaches

The goal of these therapies is to enhance relationships and social skills. Play-based therapy and social skills training are two examples (Wilczynski, 2017).

#### 3.1.5 Pharmacological treatments

When treating certain symptoms or co-occurring disorders like anxiety or ADHD, medications may be utilized (Glidden et al., 2021).

Supplements, art and music therapy, or other complementary and alternative therapies are some of the options that some families investigate (Foss-Feig et al., 2017). To best assist each person's growth and quality of life, every treatment plan is customized and may include a mix of these strategies. A complex of those treatments frequently targets incidental symptoms including agitation, violence, “fits of rage,” and abrupt affective instability. But do athletic endeavors improve or add to the results of conventional therapy?

### 3.2 Family separation and emotional needs

The separation of autistic people from their families is a major source of worry. Many people reside in government-run institutions, with caretakers from many ethnic backgrounds. While these caregivers provide vital support, the loss of parental attention throughout critical developmental stages might have long-term emotional consequences. It is critical to note that people with autism, regardless of age, frequently have unmet emotional needs from their childhood owing to this disconnection.

### 3.3 Challenges faced by parents

Unfortunately, many parents of children with autism are too busy or lack the necessary information to care for their children at home. As a result, they often have no alternative but to send their children to government-provided healthcare homes. This situation prevents these children from experiencing a typical childhood, leading to behaviors that are more characteristic of younger children, even as they grow older. They often suffer from various mental health issues and may be unable to communicate their needs and wishes effectively.

### 3.4 Behavioral problems and emotional needs

The inability to express oneself can lead to harmful conduct against others, particularly those who remind them of their parents or siblings who live at home. To address these challenges, it may be important to review their early memories and include them in activities that are appropriate for their interests and developmental stages. By doing so, they may feel more understood and responsive to direction, similar to a tiny child.

### 3.5 Benefits of physical exercise for ASD

It has been found out that when typically developing children are compared to those with ASD, the latter group exhibits generally less activity (Pan, 2008). Furthermore, it has been noted that individuals with ASD are particularly at risk due to their sedentary lifestyle, which raises the possibility of heart disease, diabetes, and obesity (Dhaliwal et al., 2019). Exercise is probably a good way to prevent these issues in the general population, and it probably works in the population with ASD as well. It has been demonstrated that a walking program improved the physical state of 10 teenagers with severe autism while simultaneously lowering their BMI index (Pitetti et al., 2007).

More importantly, with regard to the presumed positive effects on the three main problem domains of ASD (Massion, 2006), it was discovered that aerobic exercise not only improved flexibility and balance (Yilmaz et al., 2004), but it also decreased the stereotypical behavioral patterns and self-stimulation behaviors of children with ASD. Positive impacts on academic engagement (Nicholson et al., 2011), social behavior (Pan, 2010), communication skills (Hameury et al., 2010), and sensory skills (Bass et al., 2009) were documented by other investigations.

Over the past 20 years, there has been an increase in interest in the possible health benefits of physical exercise, even in spite of the general effectiveness of traditional therapies for ASD. Nonetheless, systematic study in this field is still quite rare and typically involves modest participant numbers. Overall, the data available up to 1998 painted a picture that showed that exercise not only helps individuals with ASD with their physical health but also with their maladaptive behavioral patterns (Lancioni and O'Reilly, 1998).

Better Motor Skills; Consistent exercise improves balance, motor coordination, and general physical health (Ji et al., 2023). Behavioral Improvements; Research has demonstrated that exercise reduces stereotyped behaviors as well as maladaptive behaviors like hyperactivity and hostility (Sefen et al., 2020; Toscano et al., 2022). Social Skills; Playing sports and group activities can help with communication and social skills (Toscano et al., 2022). Mental Health; Engaging in physical activity can help to improve general mental health by lowering anxiety and sadness (Riis et al., 2024). Effects on the Usage of Medication Reducing the need for medicine is another benefit of frequent physical activity. Exercise can help control symptoms of ASD without the need for medication, as it is a non-pharmacological intervention (Ji et al., 2023; Sefen et al., 2020). This is especially advantageous since it reduces the negative effects of taking medications for an extended period of time.

### 3.6 Various forms of beneficial exercise

For people with ASD, different types of physical activity can have different impacts, each with its own advantages. Every form of exercise has unique benefits, and the selection of an activity may be customized to meet the needs and preferences of the individual. People with ASD can benefit greatly from a wide range of physical activities that assist their social, emotional, and physical development.

Martial arts known to enhance attention, self-control, and motor abilities (Sefen et al., 2020). Learning martial arts, such taekwondo or karate, may help with self-control, discipline, and concentration. Repetitive motions and regimented schedules are common in these hobbies, which might be very helpful for those with ASD. A longer attention span, improved motor coordination, and a decrease in aggressive tendencies are the results (Bahrami et al., 2016).

Swimming improves balance and offers sensory advantages. Swimming has advantages for the body and the senses. The water's resistance helps improve muscle strength, while the sensory stimulation from the water may be relaxing. Benefits include enhanced sensory processing, less anxiety, and improved coordination (Pan, 2010).

In yoga, physical postures are combined with breathing exercises and relaxation methods. It can support the development of balance, flexibility, and awareness. Benefits include less tension and anxiety, better emotional control, and more bodily awareness (Rosenblatt et al., 2011).

Structured Exercise Plans; Customized plans that include a range of physical activities have the potential to be very successful (Sefen et al., 2020). Running, cycling, and team sports are just a few of the activities that these programs can incorporate. They are frequently customized to meet the requirements and skills of the person. Results include less stereotyped behaviors, enhanced social skills through group activities, and general physical health (Lang et al., 2010).

#### 3.6.1 Horseback riding (hippotherapy)

Riding a horse helps enhance muscular tone, balance, and coordination. Riding a horse has a rhythmic motion that may also be relaxing. Benefits include less anxiety, better social interaction, and increased motor abilities (Gabriels et al., 2012).

#### 3.6.2 Community-based sports

Playing sports like basketball or soccer can aid in the development of communication and collaboration skills in people with ASD. Benefits include enhanced cooperative abilities, enhanced physical fitness, and improved social connections (Healy et al., 2018).

Structured exercise reduced stereotypy by 20–30% (Sefen et al., 2020), while antipsychotics showed comparable efficacy but higher metabolic risks (Politte et al., 2015). Incorporating physical activities into daily routines for those with autism is beneficial for improving their general health and wellbeing. These activities provide you with an organized and pleasurable approach to significantly enhance a number of areas of your life (Table 1).

- Percentage improvement values reflect pooled estimates from cited randomized controlled trials. Where ranges are given, they represent variability across studies with different intervention durations (8–24 weeks). *PDMS-2*: Peabody Developmental Motor Scales, *SRS-2*: Social Responsiveness Scale
- *BOT-2*: Bruininks-Oseretsky Test of Motor Proficiency, *CBCL*: Child Behavior Checklist

**Table 1:** Benefits of physical activities for people with autism.

**Type of activities**	**Benefit**	**Description**	**Percentage improvement**	**Measurement scale**	**References**
Swimming, martial arts, dance	Improved motor skills	Enhances coordination, balance, and overall motor skills	20–30%	PDMS-2, BOT-2	Bahrami et al., 2016; Zborowska, 2024; Pan, 2010
Running, cycling, aerobics	Better cardiovascular health	Promotes heart health and helps maintain a healthy weight	15–25%	6-Min Walk Test	Zborowska, 2024; Sefen et al., 2020
Yoga, walking, swimming	Reduced anxiety and stress	Physical activity can help reduce anxiety and panic attacks	25–35%	SRS-2, CBCL	Zborowska, 2024; Pan, 2010
Team Sports (e.g., Basketball, Soccer), Group Classes	Enhanced social interaction	Improves social interaction and communication abilities	10–20%	SRS-2, SSIS	Sefen et al., 2020; Healy et al., 2018
Martial arts, yoga, dance	Improved cognitive function	Supports better processing of information, learning, memory, and retention	15–25%	BRIEF, DAS-II	Zborowska, 2024; Sefen et al., 2020; Bahrami et al., 2016
Swimming, martial arts, aerobics	Behavioral improvements	Leads to better behavior by reducing repetitive behaviors like body rocking and arm flapping	20–30%	CBCL, BRIEF	Sefen et al., 2020; Healy et al., 2018
Various physical activities	Overall quality of life	Enhances overall quality of life by improving physical, mental, and emotional wellbeing	20–30%	PedsQL, QoL-Q	Zborowska, 2024; Sefen et al., 2020

Exercise is a potential way to manage symptoms of ASD without heavily relying on medication, and it also helps the physical and mental health of those with the disorder. In the other word for those with ASD, incorporating physical exercise into their daily routine offers a potential way to manage symptoms without heavily depending on medication. It also helps their emotional and physical wellbeing.

Swimming improves aquatic skills (Pan, 2010) and reduces anxiety (*p* < 0.01) in children with ASD. Martial arts enhance attention span by 15–25% vs. controls (Bahrami et al., 2016). Clinicians should consider sensory preferences when prescribing activities (e.g., water-averse patients may tolerate martial arts better).

### 3.7 Medication's effects on autism patients

Medications are frequently used to treat irritability, anger, anxiety, and hyperactivity-symptoms linked to ASD. The following list of regularly prescribed drugs, along with their side effects:

### 3.8 Antipsychotics

Medications like risperidone (Risperdal) and aripiprazole (Abilify) are approved for treating irritability and aggression in children with ASD. These medications can help reduce challenging behaviors and improve overall functioning (Poling et al., 2017). Antipsychotics increase metabolic syndrome risk (OR 2.1; 95% CI 1.4–3.2) after 12 months of use (Politte et al., 2015). We recommend quarterly metabolic panels for patients on risperidone beyond 6 months, though this exceeds current guideline requirements.

### 3.9 Selective serotonin reuptake inhibitors (SSRIs)

SSRIs are used to treat anxiety and obsessive behaviors. Examples of SSRIs are sertraline (Zoloft) and fluoxetine (Prozac). Although they may have unfavorable side effects such increased anxiety and gastrointestinal problems, they can be useful in lowering these symptoms (Mahatmya et al., 2008).

### 3.10 Stimulants

Methylphenidate (Ritalin), a stimulant used to treat ADHD symptoms in people with ASD, can decrease hyperactivity and increase attention. But they can also result in elevated blood pressure, a faster heartbeat, and a worsening of tics and anxiety (Aishworiya et al., 2024).

### 3.11 Medication's negative effects on individuals with autism spectrum disorder

Despite their potential benefits, pharmaceuticals can have negative side effects, particularly if used often or in conjunction with other treatments. Although many symptoms of ASD can be effectively managed with medication, there are substantial risks associated with treatment, including the possibility of adverse consequences. It is essential to take these side effects into account, particularly since many people with ASD use medications for an extended period of time.

SSRIs and antipsychotics in particular are frequently given to treat behavioral and emotional problems in people with ASD. But occasionally, these drugs' negative effects might outweigh their positive advantages. Antipsychotics, for example, can cause significant weight gain and metabolic problems even while they are good at lowering agitation and aggressiveness. Prolonged usage of antipsychotics can cause serious adverse effects such metabolic syndrome, tardive dyskinesia, and weight gain. The quality of life and general health of people with ASD may be significantly impacted by these adverse effects (Aishworiya et al., 2024).

This impacts people's social connections and self-esteem in addition to their physical health. Similarly, SSRIs might worsen the symptoms they are supposed to address by increasing agitation and gastrointestinal issues, even if they are useful in treating anxiety and repetitive behaviors. Agitation, elevated anxiety, gastrointestinal problems, and weight fluctuations are some of the negative effects that SSRIs can induce. Sometimes, these side effects worsen the symptoms that they are meant to cure, creating a challenging situation for treatment (Mahatmya et al., 2008).

Furthermore, using numerous drugs, also known as polypharmacy, is prevalent among people with ASD, especially those who also have co-occurring disorders. Drug interactions and side effects, such oversedation and disorientation, are more likely as a result. These adverse consequences may have a major negative influence on an ASD person's quality of life and day-to-day functioning.

People with ASD frequently use many drugs, particularly if they also have co-occurring disorders. This raises the possibility of medication interactions and side effects, including excessive sedation, disorientation, and falls (Aishworiya et al., 2024). Chronic use of mental health drugs may result in more serious health conditions such diabetes, metabolic syndrome, and heart difficulties. These hazards emphasize how crucial it is to balance the advantages and possible disadvantages of medication as a therapeutic option for people with ASD (Aishworiya et al., 2024).

A detailed summary of the most often recommended drugs for people with autism spectrum disorder (ASD) may be found in Table 2. It draws attention to the advantages as well as any possible drawbacks, taking long-term usage into account.

**Table 2:** Advantages and adverse reactions to medication for autism patients, including extended-term issues.

**Medication type**	**Benefits**	**Short-term harmful effects**	**Long-term harmful effects**	**References**
Antipsychotics	Reduces irritability, aggression, and self-injurious behaviors	Weight gain, sedation, tremors, movement disorders, increased risk of diabetes	Increased risk of cardiovascular disease, diabetes, movement disorders, and potential loss of effectiveness	Mahatmya et al., 2008; Politte et al., 2015; Purkayastha et al., 2015; Aishworiya et al., 2024
SSRIs (Selective Serotonin Reuptake Inhibitors)	Helps reduce repetitive behaviors, anxiety, irritability, and aggression	Nausea, insomnia, increased appetite, weight gain, sexual dysfunction	Potential for increased risk of osteoporosis, cognitive decline, and loss of effectiveness	Politte et al., 2015; Baribeau and Anagnostou, 2014
Stimulants	Improves attention and reduces hyperactivity and impulsivity	Insomnia, decreased appetite, weight loss, increased cardiovascular complications, potential for abuse	Increased risk of cardiovascular issues, dependency, and potential loss of effectiveness	Baribeau and Anagnostou, 2014; Politte et al., 2015
Anti-anxiety medications	Reduces anxiety and panic attacks	Drowsiness, dizziness, dependency, withdrawal symptoms	Higher risk of dependency, withdrawal symptoms, cognitive impairment, and potential loss of effectiveness	Politte et al., 2015; Baribeau and Anagnostou, 2014
Anti-seizure medications	Controls seizures and may help with mood stabilization	Drowsiness, dizziness, liver damage, weight gain, potential for serious side effects	Increased risk of liver damage, cognitive decline, and potential loss of effectiveness	Politte et al., 2015; Baribeau and Anagnostou, 2014

This table emphasizes how crucial it is to carefully weigh the advantages and possible drawbacks of pharmaceuticals for people with autism, particularly in the long run. To guarantee the greatest results, healthcare professionals must regularly assess patients and modify treatment programs as needed.

## 4 Discussion

### 4.1 Key findings

#### 4.1.1 Synthesis of exercise vs. medication trade-offs

While risperidone rapidly reduces aggression, martial arts sustain long-term gains with fewer side effects (Toscano et al., 2022).

#### 4.1.2 Clinical recommendations

Drugs have the ability to relieve symptoms quickly, which is very helpful in emergency circumstances where prompt medical attention is required. For instance, antipsychotics such as aripiprazole and risperidone can help children with ASD exhibit less extreme irritability and aggressiveness (Poling et al., 2017). Some people's overall quality of life can be improved by SSRIs by helping to regulate anxiety and repetitive behaviors (Mahatmya et al., 2008). This instant impact can be critical for patients experiencing severe symptoms, allowing them to fully participate in everyday activities and therapeutic programs. These drugs may play a critical role in stabilizing behaviors that might otherwise impair social interactions and day-to-day functioning.

Medication has many advantages, but it also has considerable hazards. Long-term usage of antipsychotics can result in serious adverse effects such weight gain, metabolic syndrome, and mobility difficulties. These side effects have an influence on not only physical health but also emotional wellbeing and self-esteem. Furthermore, polypharmacy raises the risk of medication interactions and side effects, such as oversedation and disorientation, which can have a major impact on quality of life. The dependence on medicine can also lead to dependency, in which patients and caregivers believe that medication is the sole option, perhaps ignoring alternative effective approaches. Serious adverse effects, including weight gain, metabolic syndrome, and movement abnormalities such tardive dyskinesia, can result with long-term usage of antipsychotics (Aishworiya et al., 2024). SSRIs have the potential to worsen the symptoms they are intended to treat by increasing anxiety, causing gastrointestinal problems, and changing body weight (Mahatmya et al., 2008). Furthermore, polypharmacy raises the possibility of medication interactions and side effects, such disorientation and oversedation, which can have a serious negative influence on quality of life (Aishworiya et al., 2024).

Physical activity has multiple advantages with few adverse effects. Regular exercise can boost motor abilities, improve social connections, and alleviate anxiety and despair. Swimming, yoga, and martial arts have been demonstrated to enhance coordination, balance, and general physical fitness, all of which are common challenges for people with ASD. Furthermore, physical exercise can be used as a non-pharmacological therapeutic, minimizing the need for medicine and accompanying dangers. Physical activities can also create a sense of accomplishment and enhance self-esteem, so improving general mental health. Frequent exercise can lessen anxiety and sadness, improve social connections, and improve motor abilities (Lang et al., 2010). Exercises that have been demonstrated to enhance balance, coordination, and general physical fitness include yoga, martial arts, and swimming. These are areas that people with ASD frequently struggle with (Pan, 2010; Rosenblatt et al., 2011). Furthermore, exercise can work as a non-pharmacological intervention, lowering the risk and requirement for medication (Aishworiya et al., 2024).

The basic problems of physical activity are the need for time, desire, and resources to sustain a consistent exercise practice. Individuals with ASD may require structured and supervised programs to guarantee safety and efficacy, which can be resource-intensive. To maintain safety and efficacy, individuals with ASD may need supervised, organized programs, which might demand a lot of resources (Lang et al., 2010). Furthermore, some people may have physical restrictions or sensory sensitivity issues that make particular forms of exercise difficult.

According to the research, encouraging physical exercise is the best way to improve the health of people with autism, with medication being used as a supplemental strategy when needed. Exercise need to be suggested by medical experts as a component of an autistic patient's therapy regimen.

Customizing exercise plans to a person's interests and physical capabilities can increase efficacy and participation. For instance, adding enjoyable activities like martial arts or swimming might boost adherence and motivation (Pan, 2010; Rosenblatt et al., 2011).

The findings imply that physical exercise should be advocated as a main strategy of improving health in people with autism, with medication given as a supplement when necessary. Healthcare practitioners should consider promoting exercise as part of their treatment strategy for people with autism. Promoting the incorporation of physical activity into daily routines can aid in the long-term adoption of exercise as a lifestyle choice. According to Lang even basic pursuits like walking, playing in the park, or taking part in neighborhood sports can have a positive impact (Lang et al., 2010).

Tailoring exercise regimens to each person's interests and skills can increase participation and efficacy. For example, including activities that the person loves, such as swimming or martial arts, might boost motivation and adherence. Personalized training can also treat particular motor skill weaknesses and offer focused advantages.

Encouraging the incorporation of physical activity into daily routines can help make exercise a long-term part of life. Simple activities such as walking, playing in the park, and engaging in community sports might be beneficial. Making physical activity a regular part of your day might help you develop good habits and routines.

Adequate assistance and monitoring are essential, especially for people who have severe symptoms or physical restrictions. Trained specialists, such as physical therapists or specialized trainers, can assist in designing and monitoring exercise programs to guarantee their safety and efficacy. Support from family members and caregivers can also help to retain motivation and consistency.

To guarantee safety and efficacy, qualified specialists like physical therapists or certified trainers can assist in creating and supervising exercise regimens (Aishworiya et al., 2024). When physical exercise is not enough to address acute symptoms, medications should be taken. For example, medication can offer the stability required to enable an individual to engage in physical activities in situations including significant hostility or self-injurious conduct (Poling et al., 2017).

Medications should be used to treat acute symptoms or when physical exercise alone is ineffective. For example, in situations of acute violence or self-injurious conduct, medicines can give the stability required to allow the individual to engage in physical activities. This balanced strategy can aid in maximizing the effects of both therapies.

To achieve the best results, medication and physical activity regimens must be monitored and adjusted on a consistent basis. Healthcare practitioners should collaborate closely with patients and their families to review drug efficacy and side effects and make any required changes. This continual review ensures that the treatment plan is still effective and tailored to the individual's needs. Based on my professional experience, I've seen that many persons with autism do not require further medication. Instead, decreasing drugs and increasing physical exercise may be beneficial. Physical activities benefit not just physical health but also emotional wellbeing and social interaction, allowing for a more holistic approach to ASD care.

### 4.2 Future research

#### 4.2.1 Advice for care

We should change our emphasis from boosting medicine to encouraging greater physical activity and mental support *Hybrid models (exercise* + *low-dose medication) and microbiota-focused interventions warrant RCTs*. Individuals with autism ought to be treated with the same care and respect as other children. Even when their children have mental health issues, parents are generally hesitant to give them too much medicine. Similarly, we should avoid overmedicating people with autism and instead encourage activities that promote their general growth and wellbeing. Physical activity benefits not just physical health but also mental wellbeing and social connection. Addressing the emotional needs of people with autism, especially those who have been removed from their family, is also critical. Providing enough emotional support and chances for social engagement can help alleviate some of the difficulties associated with ASD while also improving their overall development and quality of life. Institutionalized children with ASD show 2.3 × higher emotional dysregulation rates vs. family-raised peers (*p* < 0.001; Green et al., 2009). Early reintegration programs should be prioritized, even when parental training resources are limited.

#### 4.2.2 Suggestions for future research and policy

Future study should investigate the long-term impacts of physical activity and how it might be included into treatment regimens for people with autism. Researchers should look at the exact sorts of physical activities that are most effective for different age groups and degrees of competence on the autistic spectrum. Furthermore, studies should look at the influence of physical exercise on gut microbiota composition and the following impacts on neurobehavioral disorders.

Healthcare practitioners should urge patients to include regular exercise into their daily regimen. This can be accomplished by creating personalized fitness routines tailored to each person with autism's interests and skills. Caregivers and educators should get training and materials to help them execute these initiatives. Furthermore, rules should be implemented to guarantee that people with autism receive the emotional support they require, preferably in a family environment. This involves giving families with tools and assistance so they may care for their children at home, decreasing the need for institutionalization. Policies should also encourage caregiver training to address the specific emotional and developmental needs of people with autism. By putting physical exercise and emotional support first, we can build a more holistic and successful approach to autism treatment and growth. Exercise and medication show comparable efficacy for aggression reduction at 12 weeks (*p* = 0.34), but exercise maintains effects longer (Toscano et al., 2022). Future research should explore hybrid models (e.g., exercise + low-dose medication) for treatment-resistant cases.

## 5 Conclusion

In conclusion, while drugs can give immediate symptom relief and be successful in addressing certain behavioral difficulties, they also carry considerable hazards and the potential for long-term health concerns. Physical activity, on the other hand, has several advantages with little negative effects and should be promoted as the primary approach of improving health in people with autism. Healthcare practitioners can assist people with ASD improve their overall quality of life by incorporating physical exercise into their daily routines and utilizing pharmaceuticals as a supplement when necessary. Furthermore, addressing the emotional needs and family dynamics of people with autism is critical for their long-term health. Ensuring that these people receive enough emotional support and chances for social engagement can help alleviate some of the difficulties associated with ASD and improve their overall development and wellbeing. Prioritizing physical activity and emotional support can reduce medication dependence in ASD. Future work should integrate biomarker monitoring (e.g., microbiota) with behavioral outcomes to personalize treatment.
